# The development of an online implant manufacturer application: a knowledge-sharing platform for the Swedish Hip Arthroplasty Register

**DOI:** 10.1080/17453674.2019.1608094

**Published:** 2019-04-30

**Authors:** Johanna Vinblad, Daniel Odin, Johan Kärrholm, And Ola Rolfson

**Affiliations:** a The Swedish Hip Arthroplasty Register, Centre of Registers Västra Götaland;; b Department of Orthopedics, Institute of Clinical Science, Sahlgrenska Academy, University of Gothenburg, Sweden

## Technique

### Data source

SHAR (founded in 1979) is a national quality register covering all orthopedic units performing hip replacement surgery in Sweden. The completeness of registrations during the last 10 years varies between 97–98% for total hip replacements, 95–97% for hemi-arthroplasties, and 91–95% for revision procedures when linking data to the Swedish national patient register (Kärrholm et al. 2018). The register currently covers more than 360,000 hip procedures, 1,100,000 registered items and 6,100 unique components. Administrative staff at the units report surgical variables for primary procedures and reoperations. Medical records copies covering admission, surgical procedure, and discharge for all patients undergoing reoperations are sent to the register for further extraction of data. 57 variables are collected regarding the primary surgery and 99 variables for reoperations. The register also retains a separate component database with variables describing attributes of the implant. The component database contains 141 variables. Additionally, the register comprises 54 variables collected through the patient-reported outcome measures program preoperatively, and 1, 6, and 10 years after surgery. Patient-reported outcome measures are all maintained in the principal database. Lastly, the register contains a separate surgical environment database with hospital-level aggregate information.

For the development of the manufacturer application, we included variables related to diagnosis, component features, reason for surgery, and surgery outcome in terms of revisions. Patient-reported outcome measures and surgical environment variables were not included.

### Work process

The project delivery model can be divided into 7 phases (Figure). Planning, development, and test were carried out in iterative cycles to optimize product outcome.

**Figure:**
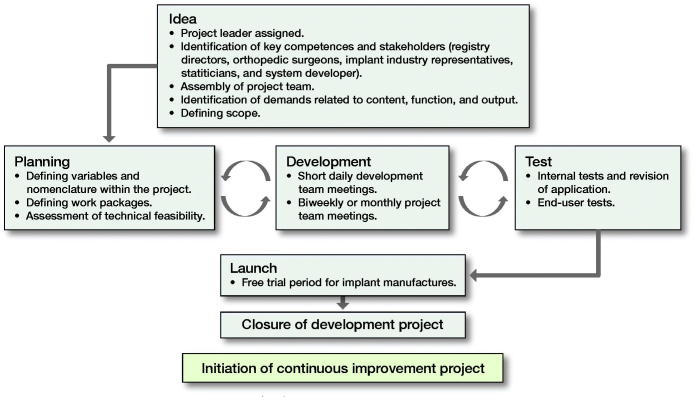
The project delivery model.

### Platform specification

The design of the application is based on the IT platform new Stratum and scripts for the R free software environment (https://www.r-project.org/) for statistical computing and graphics. Stratum is a technical platform for describing, collecting, and presenting data from quality registers in healthcare developed by the Centre of Registers, Västra Götaland. The platform provides a register and its users with a range of features for continuous quality improvement and follow-up, including advanced form management, statistics engine (in R), and visualization support (user interface components from ExtJS, developed by Sencha [https://www.sencha.com/]).

### Validation process

The validation was done in several steps. The back-end code, which was written in R, was reviewed by 2 statisticians. Repeated quality assurance tests were performed. Comparisons were carried out between raw data and R-script outcome as well as between outcome of the R-scripts and outcome in the interface. Finally, the interface was tested by several persons not involved in the development of the project to find inconsistencies.

### Access/login requirements

User access to data is restricted. The application displays only data on implants for the specific company, which the user represents. An electronic personal identification system widely used in Sweden, Mobile BankID, is used to confirm identity and securely log in to the application.

### Restriction of data usage

In order to access the application, a contract must be in place between the industry and the register. This agreement restricts the use of data. The analyses performed may be used within the company for internal use, for regulatory purposes, and for marketing purposes as reported to regulatory agencies.

The SHAR charges an annual subscription fee for the service based on number of implants registered during the previous year in addition to a fixed basic rate.

## Product description

Using previous experience from industry collaboration in combination with discussions between industry and registry as well as in-house orthopedic expertise, key areas were identified. These were consolidated into 4 modules in the application: volume, revised implants, implant survival, and market share. The 4 modules will be further discussed below, and a summary of the choices and filters can be accessed in Tables 1–4, see Supplementary data.

### Volume

Broken down by hospitals, the volume module displays number of implants registered based on catalogue number for a selected period. There are several data-filtering options available. Type of surgery is one of them, allowing for filtering with regards to primary or revision surgery. Other filtering opportunities are type of prosthesis and group of implant. The date range is variable between 1999 and current date with 28 days being the minimum number of days that you can choose (Table 1, see Supplementary data).

### Revisions per implant

The 2nd module presents number of inserted and revised implants on article number level in Sweden. The filtering option “Type of surgery” allows the user to explore revisions after primary surgery and/or all re-revisions after revision surgery. Search criteria allow for selection of either total or hemi-arthroplasty as well as implant family as previously defined by the register. It is also possible to select type of revision; all 1st-time revisions, 1st stem revision, 1st cup revision, and 1st revision of other kind. For example, if 1st stem revision is selected, then any revision that is not a stem revision will be disregarded. In this module, there is also a possibility to focus on cause for revision. The returned revision data will be divided into revision occurring 0–90 days, 91 days to 2 years, and more than 2 years after the surgery. The date range may be set between any time point from 1999 to current date and the shortest date range is 1 year (Table 2, see Supplementary data).

### Implant survival

The 3rd module displays Kaplan–Meier and cumulative incidence survival graphs for stems and/or cup families based on 1st revision after primary surgery. The survival graphs can be tailored for specific needs by choosing patient population based on diagnosis, type of prosthesis, type of revision, and cause for revision (Table 3, see Supplementary data). In this module, the stems and cups are grouped, since there are usually not a sufficient number of observations related to a specific article number (e.g., a specific cup design with a specific size) to perform a robust analysis. On the other hand, if a specific cup design is selected for analysis all article numbers included (e.g., sizes) can easily be extracted.

It is possible to choose 1 or more implant families to be analyzed in 1 group. A specified group of company stems can be combined with a corresponding group of cups without restrictions. It is also possible to combine a specified group of stems with all company cups and vice versa and compare combinations of stems and cups from one’s own company with aggregated data on hip prostheses from all other suppliers. A number of revision outcomes can be defined such as all 1st-time revisions, 1st stem revision, 1st cup revision, and other types of 1st-time revisions. The Kaplan–Meier analyses provide estimates of the probability of a selected implant or implant group being revised at given time points. Competing risk-based cumulative incidence, on the other hand, will visualize the proportion of implants that have been revised and the proportion of patients who have died at given time points. The date range may be set between any time point from 1999 to current date and the shortest date range is 1 year. The primary surgeries performed in the chosen time interval are followed up until current date or until the number of hips at risk is below 50. In addition, 95% confidence intervals are visualized in the graphs.

### Market share

The last module in the application addresses market share for a selected type of implant (i.e. cup, stem, head, liner, and distal plug) from the company. It is also possible to filter with regards to type of prosthesis (i.e., total or hemi arthroplasty) and fixation. In addition to market share, manufacturers may also access the total number of registered implants in Sweden as compared with all implants used for the specific company (Table 4, see Supplementary data).

### Outputs

All aggregated results are presented in tables and graphs may be downloaded to excel.

## Discussion

The overall aim with quality registers is to sustain and improve healthcare for patients. The Swedish Hip Arthroplasty Register is working with a wide group of stakeholders in order to ensure delivery of high-quality healthcare. Strong collaboration between the registry and the industry is paramount. Early detection of implants with substandard performance is important for the industry, the healthcare system and patients. There are values in the form of saving patient suffering as well as a high economic value in detecting failing implants early on.

Several ongoing international initiatives aim to monitor and assess implants survival, such as the Orthopaedic Data Evaluation Panel (ODEP, http://www.odep.org.uk/), Beyond Compliance (http://www.beyondcompliance.org.uk/), and Arthroplasty Watch (http://www.arthroplastywatch.com/). The consequences of using an evidence-based system for rating of implants in the UK are demonstrated by Ng Man Sun et al. (2013). That paper highlights that healthcare providers in the UK follow recommendations based on clinical evidence regarding choice of implants. The Australian Orthopaedic Association National Joint Replacement Registry (AOANJRR) has previously reported a method of detecting prostheses with a higher than expected rate of revision, so-called “outlier” prostheses (de Steiger et al. 2013).

The coexistence of several robust systems for detecting failing implants should increase the likelihood of as early detection as possible.

### Limitations

Early detection of failing implants or product development may require different statistical robustness of data. In some cases, one would wish to access data as early as possible to look at trends and for other activities, and the data must be solid and robust. When discussing this, patient data security is also an important factor. We have included time-range restrictions and minimal number of hips at risk to allow for any analysis in order to ensure patient confidentiality as well as data robustness.

In any real-time search application it is important to keep in mind the register validation process. At the beginning of a new year the data going into the annual report for the previous year are validated in several steps to ensure high quality. During the year, administrative staff at the hospital carry out registrations. The time between surgery and registration will to a certain extent differ between hospitals, which means that there is an inherent uncertainty in analyses based on aggregated data collected during the last weeks. When the annual report has been published for a specific year, the data can be regarded as well validated up to this specific year. Until then the data available from the latest calendar year should be considered as preliminary data.

When comparing data generated in the application one should consider that patient population characteristics, for example age, sex, and diagnosis, might differ between data sets and this could potentially influence the results.

In conclusion, the sharing of data between register and manufacturer comes with a responsibility. The manufacturers must be aware of limitations and take the responsibility when presenting the data. There is a critical balance between early access to data with the intention to alert regarding failing implants, and delivering robust high-quality data. All relevant stakeholders must be aware of this and use the data appropriately. This is partly addressed in the contract as data can only be used for marketing purposes as reported to the regulatory agency.

To summarize, a well-established collaboration between the registry and the industry is not only beneficial for industry but also for the register, the orthopedic profession and not least the patient. Poorly performing implants can indeed be identified without involvement of the industry, but we think that this application will increase their interest in this process. We hope that our newly developed application will stimulate a collaboration to find the true background behind substandard implant performance, which may or may not be related to the properties of the prosthesis or the implant part of interest itself. The registry will continue to build on the application and will continue with yearly meetings with the industry in order to share knowledge and develop the collaborations further.

## Supplementary Material

Supplemental Material
