# Supplementary material for: The development of an online implant manufacturer application: a knowledge-sharing platform for the Swedish Hip Arthroplasty Register
Source: Acta Orthop. 2019 Apr 30;90(4):406–9. doi: 10.1080/17453674.2019.1608094 (PMC6718186; doi:10.1080/17453674.2019.1608094)
Supplement: Supplemental Material [file IORT_A_1608094_SM5180.pdf]

Supplementary data

Table 1. Module 1: Volume

Unit	Type of surgery	Type of prosthesis	Type of implant	Item
All units	All<br>Primary<br>Revision	All<br>Total<br>Hemi-	All<br>Head<br>Cup<br>Liner<br>Stem<br>Plug<br>Dual mobility liner	All catalogue numbers

Table 4. Module 4: Market share

Region	Type of prosthesis	Type of implant	Fixation
All regions	All<br>Total<br>Hemi-	All<br>Head<br>Cup<br>Liner<br>Stem<br>Plug<br>Dual mobility liner	All<br>Non-cemented<br>Cemented<br>Hybrid<br>Reverse hybrid<br>Resurfacing<br>Not specified

Table 2. Module 2: Revision

Type of surgery	Type of prosthesis	Type of revision	Type of implant	Item	Cause for revision
Primary<br>Revision	Total<br>Hemi-	All 1st-time revisions<br>1st stem revision<br>1st cup revision<br>1st revision of other kind	All<br>Head<br>Cup<br>Liner<br>Stem<br>Plug<br>Dual mobility liner	All catalog numbers	All<br>Aseptic loosening<br>Deep infection<br>Dislocation<br>All aseptic causes

Table 3. Module 3: Implant survival

Diagnosis	Type of prothesis	Stem	Cup	Type of revision	Cause	Calculation method
All<br>Primary osteoarthritis<br>Inflammatory joint disease<br>Acute trauma, hip fracture<br>Sequelae childhood hip disease<br>All aseptic causes<br>Femoral head necrosis<br>Sequelae trauma/fracture<br>Tumor<br>Other secondary osteoarthritis<br>Acute trauma, other<br>Other	Total<br>Hemi-	Company stems<br>Other manufacturer	Company cups<br>Other manufacturer	All 1st-time revisions<br>1st stem revision<br>1st cup revision<br>1st revision of other kind	All<br>Aseptic loosening<br>Periprosthetic infection<br>Dislocation	Kaplan–Meier<br>Cumulative incidence
